# Right ventricular load and contractility in HIV-associated pulmonary hypertension

**DOI:** 10.1371/journal.pone.0243274

**Published:** 2021-02-23

**Authors:** Arun Rajaratnam, Sofiya Rehman, Prerna Sharma, Vikas K. Singh, Melissa Saul, Rebecca R. Vanderpool, Mark T. Gladwin, Marc A. Simon, Alison Morris

**Affiliations:** 1 Pittsburgh Heart, Lung, Blood and Vascular Medicine Institute, University of Pittsburgh, Pittsburgh, PA, United States of America; 2 Department of Medicine, University of Pittsburgh, Pittsburgh, PA United States of America; 3 Analytics Center, University of Pittsburgh, Pittsburgh, PA, United States of America; 4 Department of Bioengineering, University of Pittsburgh, Pittsburgh, PA, United States of America; 5 Division of Pulmonary, Allergy and Critical Care Medicine, University of Pittsburgh, Pittsburgh, PA, United States of America; 6 Division of Cardiology, University of Pittsburgh, Pittsburgh, PA, United States of America; 7 UPMC Heart and Vascular Institute, Pittsburgh, PA, United States of America; Vanderbilt University Medical Center, UNITED STATES

## Abstract

**Background:**

People living with human immunodeficiency virus (PLWH) are at risk of developing pulmonary hypertension (PH) and right ventricular (RV) dysfunction, but understanding of the relationship of RV function to afterload (RV-PA coupling) is limited. We evaluated the clinical and hemodynamic characteristics of human immunodeficiency virus (HIV)-associated PH.

**Methods:**

We performed a retrospective review of patients with a diagnosis of HIV undergoing right heart catheterization (RHC) from 2000–2016 in a tertiary care center. Inclusion criteria were diagnosis of HIV, age ≥ 18 years and availability of RHC data. PH was classified as either pulmonary arterial hypertension (PAH; mean pulmonary arterial pressure [mPAP] ≥ 25mmHg with pulmonary artery wedge pressure [PAWP] ≤ 15mmHg) or pulmonary venous hypertension (PVH; mPAP ≥ 25mmHg with PAWP > 15). We collected demographics, CD4 cell count, HIV viral load, RHC and echocardiographic data. The single beat method was used to calculate RV-PA coupling from RHC.

**Results:**

Sixty-two PLWH with a clinical likelihood for PH underwent RHC. Thirty-two (52%) met PH criteria (15 with PAH, 17 with PVH). Average time from diagnosis of HIV to diagnosis of PH was 11 years. Eleven of 15 individuals with PAH were on antiretroviral therapy (ART) while all 17 patients with PVH were on ART. Compared to PLWH without PH, those with PH had an increased likelihood of having a detectable HIV viral load and lower CD4 cell counts. PLWH with PAH or PVH had increased RV afterload with normal RV contractility, and preserved RV-PA coupling.

**Conclusion:**

PLWH with PH (PAH or PVH) were more likely to have a detectable HIV viral load and lower CD4 count at the time of RHC. PLWH with PAH or PVH had increased RV afterload, normal RV contractility, with preserved RV-PA coupling suggestive of an early onset, mild, and compensated form of PH. These results should be confirmed in larger studies.

## Introduction

Human immunodeficiency virus (HIV) infection has been implicated as an independent risk factor for pulmonary arterial hypertension (HIV-PAH) [1], occurring in approximately 1 out of every 200 persons living with HIV (PLWH) [1, 2]. Studies likely underestimate the true prevalence of HIV-PAH because the disease often exists prior to the onset of symptoms and diagnosis [3, 4]. Though the exact etiology by which HIV causes PAH is unknown, 3 distinct proteins: Nef, Tat, and gp120, have been associated with vascular changes in PAH [1, 5] Pulmonary hypertension (PH) related to diastolic dysfunction can be secondary to alterations of left ventricular (LV) function that may be related to myocardial damage induced by the virus [6]. PH may become more prevalent as improved medical management have increased the life expectancy of HIV-infected patients [1].

There is a paucity of data that compare different pathophysiological phenotypes of PH (precapillary or post-capillary) in HIV and their relation to right ventricular (RV) function. PAH is characterized by the presence of increased pulmonary vascular resistance (precapillary pulmonary hypertension), resulting in RV pressure overload and eventually right heart failure. Thus, RV function is a major determinant of outcome in PAH [7, 8]. PH secondary to LV dysfunction (PVH), may be more common than PAH in patients with HIV given the relatively high presence of subclinical LV dysfunction [6]. HIV-associated LV dysfunction has been studied since HIV-related cardiomyopathy was recognized in the 1980s with an estimated prevalence of LV systolic dysfunction from 2% to 20%; however, higher rates of preserved LV function with diastolic dysfunction range from 26% to 50% [9]. These studies found an association between the extent of immunodeficiency and systolic dysfunction as well as a higher incidence and prevalence of cardiomyopathy in individuals with CD4 cell counts <400 cells/mm^3^ [9].

RV function has been strongly associated with outcomes in multiple disease states including PH and heart failure with reduced and preserved ejection fraction [10–12]. There have been few studies of RV function in individuals with HIV-related PH. One prior study noted RV dysfunction may be independent of LV function or PH in individuals without previously established PH [13]. In this study, we examined characteristics of HIV-related PH and detailed RV function parameters.

## Methods

Subjects were selected from a de-identified database of all patients who underwent a right heart catheterization (RHC) at the University of Pittsburgh Medical Center between 2000 and 2016. We selected individuals with a diagnosis of HIV (ICD-9 code: 042) in the Electronic Health Record. Patients were categorized into 2 groups: Pulmonary Hypertension (PH, defined as mean pulmonary arterial pressure [mPAP] ≥ 25mmHg) and No Pulmonary Hypertension (No PH, defined as mPAP≤ 25mmHg). PH was further sub-categorized as either pulmonary arterial hypertension (PAH; defined as mPAP ≥ 25mmHg with pulmonary artery wedge pressure [PAWP] ≤ 15mmHg) or pulmonary venous hypertension (PVH; defined as mPAP ≥ 25mmHg with PAWP > 15). Data collected included demographics, medical history, medications, CD4 cell count, and HIV viral load. We analyzed echocardiographic and cardiac catheterization data that were collected within 6 months of each other for complete LV systolic and diastolic measurements and RV systolic measurements. The IRB of the University of Pittsburgh approved the study.

### Evaluation of RHC data

RHC were performed as clinically indicated. All data were reviewed, and pressures measured at end-expiration. Cardiac output was determined by both the thermodilution and indirect Fick methods; the thermodilution cardiac output was preferentially used when available. Pulmonary vascular resistance was calculated as (mPAP–PAWP) / cardiac output. Pulmonary artery compliance (PAC) was calculated as stroke volume/pulmonary artery pulse pressure (PASP-PADP).

### Calculation of RV function parameters

RV pressure waveforms were analyzed for all right heart catheterizations. The single beat method was used in analyzing RV contractility (or end-systolic elastance, Ees) [14] An average RV pressure waveform was generated for each patient across one respiratory cycle, typically, 4–6 beats. A manual sine wave extrapolation of the early systolic and diastolic portions of the RV pressure curve was used to calculate Pmax, the theoretical pressure generated by the ventricle if isovolumetric contraction occurred. End-systolic pressure was assumed to be mPAP. Ees was calculated as the slope of end-systolic pressure volume line, Ees = (Pmax − mPAP) / (stroke volume). Arterial elastance (Ea) was estimated by mPAP / (stroke volume). RV–arterial coupling (Ees/Ea) was simplified for volumes as Pmax / mPAP– 1. Passive diastolic function of the RV was calculated as Beta [15]. Stroke volume was calculated as cardiac output measured by the Fick method during the RHC divided by heart rate and indexed to body surface area (SVI).

### Evaluation of echocardiographic data

Echocardiography was performed and analyzed in accordance with American Society of Echocardiography recommendations [16–18]. Echocardiographic data were reviewed by two independent physicians (A.R and M.A.S). RV outflow time velocity time integral (RVOT VTI) was measured by tracing the Doppler velocity signal averaged over a single beat. RV fractional area of change (FAC) was obtained by measuring RV end-diastolic (RVEDA) and end-systolic areas (RVESA) from the apical 4-chamber view [RVFAC = (RVEDA–RVESA) / RVEDA) × 100] [17]. Tricuspid annular systolic plane excursion (TAPSE) was measured by M-mode of the lateral tricuspid valve annulus.

### Statistical analysis

Normally distributed variables are shown as mean ± standard deviation and non-normally distributed variables as median with interquartile range (IQR). Categorical variables are described by percentage (number) unless otherwise stated. Between-group comparisons were made using student’s T-test for normally distributed variables, Mann-Whitney U test for non-normally distributed data, and Chi-square test for categorical variables. Correlation was calculated using Pearson’s correlation. Univariate logistic regression by Cox proportional hazards ratio was used to assess risk of PH associated with detectable HIV viral load. Survival was assessed from the date of RHC to last follow up or date of death, with Kaplan-Meir survival curves with log-rank test for comparison between groups. Statistical analysis was performed using SPSS 24 (Version 24, SPSS Inc., Chicago, IL).

## Results

### Pulmonary hypertension versus no pulmonary hypertension

A total of 62 patients were identified with HIV who underwent RHC evaluation for PH. Thirty–two (52%) met PH criteria and 30 did not have PH. The groups were predominantly male (70% and 69% in the no PH and PH groups respectively) with an average age of 51 years. There were no significant differences in age, sex, or race between groups (Table 1). Eighty-eight percent of patients with PH were on anti-retroviral therapy (ART). A large proportion of patients had past or ongoing smoking history, 69% and 57% in PH and no PH, respectively. CD4 count was significantly lower in the PH group, 374±252 cells/mm^3^ versus 594±367 cells/mm^3^ in patients without PH (P = 0.02). Patients with PH were more likely to have a detectable HIV viral load, with an odds ratio 12.0 (CI: 1–122, P = 0.04) by univariate logistic regression analysis. Vasodilator use (defined as use of calcium channel blocker, phosphodiesterase type 5 inhibitor [PDE5i], endothelin receptor antagonist, or prostacyclin) was similar between PH and no PH groups (28% vs 17%, respectively, P = 0.28). In patients without PH, 4 patients were on calcium channel blockers (CCB) and 1 patient was on a PDE5i. In the PH group, 8 out of 9 patients were on PDE5i monotherapy, with 6 patients on tadalafil and 2 on sildenafil. One patient was started on IV epoprostenol in the intensive care unit, subsequently weaned off and transitioned to combination therapy with a PDE5i and endothelin receptor antagonist. Primary presenting symptoms in the PH group were progressive shortness of breath, pedal edema, fatigue, syncope or near-syncope, and chest pain.

**Table 1:** Characteristics of PLWH with and without pulmonary hypertension.

	No Pulmonary Hypertension N = 30	Pulmonary Hypertension	P value
		N = 32	
**Age (years)**	52 (10)	51 (10)	0.64
**Female**	9/30 (30)	10/32 (31)	0.92
**African American**	13/29 (45)	15/31 (48)	0.78
**CAD**	4/30 (13)	5/32 (16)	0.80
**IVDU**	8/30 (27)	8/32 (25)	0.88
**History of Smoking**	17/30 (57)	22/32 (69)	0.33
**Hypertension**	13/30 (43)	12/32 (38)	0.64
**Vasodilator use**	5/30 (17)	9/32 (28)	0.28
**CD4 <400**	9/26 (35)	17/26 (65)	0.03
**CD4 count (cells/mm**^**3**^**)**	594 (367)	374 (252)	0.02

Results reported as number/total (total %) or mean (SD). CAD indicates coronary artery disease; CD4, cluster of differentiation 4; IVDU, Intravenous drug use.

Twenty-seven of 32 patients with PH (84%) had available echocardiographic and cardiac catheterization data (Table 2). Patients with PH had significantly worse RV function as estimated by RVFAC (36 ± 15% vs 47 ± 11%, in PH vs no PH, respectively, P = 0.03) and RVOT VTI (0.15 m [0.12–0.19 m] vs 0.20 m [0.18–0.36m], in PH vs no PH, respectively, P <0.01); however, TAPSE was not significantly different (1.9 ± 0.7 cm vs. 1.6 ± 0.3 cm, P = 0.15) in PH vs no PH group. Estimated pulmonary artery systolic pressure (ePASP) was similar in both groups (46 ± 28 mmHg vs. 38 ±15 mmHg, P = 0.22). Tricuspid regurgitant velocity (TRV) divided by RVOT VTI (TRV/RVOT VTI), a noninvasive measure of pulmonary vascular resistance (PVR) [19] had minimal but significant correlation to PVR (R^2^ = -0.058, P<0.01) when compared to PVR as determined by RHC. TRj/RVOT was not significantly correlated with Ea (R^2^ = 0.41, P = 0.06).

**Table 2:** Echocardiographic and hemodynamic characteristics of PLWH with and without pulmonary hypertension.

	No Pulmonary Hypertension	Pulmonary Hypertension	P value
	N = 30	N = 32	
**Heart rate (bpm)**	68 (13)	74 (17)	0.22
**ePASP (mmHg)**	38 (15)	46 (28)	0.22
**EF (%)**	60 (55–60)	55 (50–60)	0.07
**RVOT VTI (m)**	0.20 (0.18–0.36)	0.15 (0.12–0.19)	<0.01
**mPAAT (cm)**	137 (100–164)	128 (86–184)	0.79
**TAPSE (cm)**	1.6 (0.3)	1.9 (0.7)	0.15
**FAC (%)**	47 (11)	36 (15)	0.03
**MPAP (mmHg)**	19 (17–22)	32 (29–53)	<0.01
**PAWP (mmHg)**	11 (3.8)	18 (8.9)	<0.01
**CO (L/min/m**^**2**^**)**	4.8 (4.3–7.2)	5.0 (4.4–6.3)	0.43
**PAC (mL/mmHg)**	4.4 (3.8–6.0)	2.0 (1–0.2)	<0.01
**PVR (Woods Units)**	1.56 (1.0–0.2)	3.4 (2.2–7.0)^j^	<0.01
Ees (mmHg/mL)*	0.30 (0.23–0.54)	0.68 (0.48–1.10)	<0.01
Ea (mmHg/mL)*	0.31 (0.30–0.53)	0.79 (0.52–1.5)	<0.01
Ees/Ea*	0.88 (0.63–1.17)	0.83 (0.59–1.11)	0.61
Diastolic Stiffness Beta*	0.06 (0.10)	0.08 (0.20)	0.94

Results reported as number (%), mean (standard deviation) or median (interquartile range). CI indicates cardiac output; Ea, arterial elastance; Ees, end systolic elastance; EF, ejection fraction; ePASP, estimated pulmonary artery systolic pressure; FAC, fractional area change; mPAAT, mean pulmonary artery acceleration time; mPAP, mean pulmonary artery pressure; PAH, pulmonary arterial hypertension; PAWP, pulmonary artery wedge pressure; PAC, Pulmonary artery compliance; pulmonary vascular resistance; RVOT VTI, right ventricular outflow tract velocity time integral; TAPSE, tricuspid annular plane systolic excursion.

*Single beat analysis was able to be calculated for 18 patients in the No Pulmonary Hypertension group and 24 patients in the Pulmonary Hypertension group.

The mPAP and PVR were significantly higher in PH vs. No PH groups while cardiac output was equivocal (Table 2). PAC was lower in the PH group vs No PH group (2.0 mmHg [1.7–3.6 mmHg] vs 4.4 mL/mmHg [3.8–6.0 mL/mmHg]) in a reciprocal relationship to PVR. RV afterload as measured by pulmonary arterial elastance (Ea) was greater in PH as was RV contractility (Ees), but no significant difference was noted in RV-PA coupling (E_es_/E_a_) between groups, suggestive of a compensated physiological state. Passive diastolic stiffness of the RV (Beta) was also similar between groups.

### Pulmonary arterial hypertension versus pulmonary venous hypertension

Patients with PH (n = 32) were categorized into PAH (n = 15) and PVH (n = 17) based on RHC, with 13 patients diagnosed as PAH and 14 patients with PVH having complete echocardiographic and hemodynamic data. These subgroups were predominantly male, with an average age of 50 and 52 years in PAH and PVH, respectively. The PAH group was predominantly Caucasian (64%) while the PVH group was largely African American (63%). There were no significant differences in the age, sex, race, or CD4 count between groups (Table 3). There was a higher prevalence of hypertension in PVH (59% vs 13% in PAH, P = <0.01). Eleven of the 15 individuals with PAH were on ART while all patients with PVH were on ART.

**Table 3:** Characteristics of PLWH with pulmonary arterial hypertension versus pulmonary venous hypertension.

	Pulmonary Arterial Hypertension	Pulmonary Venous Hypertension	P value
	N = 15	N = 17	
**Age (years)**	50 (11)	52 (9)	0.58
**Female**	6/15 (40)	4 /17 (24)	0.32
**African American**	5/14 (36)	10/16 (63)	0.14
**CAD**	1/15 (7)	4/17 (24)	0.19
**IVDU**	5/15 (33)	3/17 (18)	0.31
**History of Smoking**	9/15 (60)	13/17 (77)	0.32
**Hypertension**	2/15 (13)	10/17 (59)	<0.01
**Vasodilator use**	6/15 (40%)	3/17 (18%)	0.16
**Anti-retroviral use**	11/15 (73)	17/17 (100)	0.02
**CD4 <400**	10/13 (77)	7/13 (54)	0.22
**CD4 count (cells/mm^3^)**	342 (170)	406 (319)	0.53

Results reported as number/total (total %) or mean (SD). CAD indicates coronary artery disease; CD4, cluster of differentiation 4; IVDU, Intravenous drug use.

Evaluation of RV function using echocardiography showed ePASP was elevated in both groups, with reduced RVFAC in PAH (31±13) compared to PVH (43±15), however neither variable was statistically significant. TASPE was preserved, greater than 1.8 cm in both groups suggestive of conservation of global RV function, but no differences were found by mPAAT and RVOT VTI. Based on RHC, mPAP was elevated in PAH and PVH, non-significantly, with the exception of PAWP in PVH. PVR was 3.4 Woods units (3.0–7.5 WU) versus 3.9 Woods units (1.7–5.4 WU) and PAC was 2.5mL/mmHg (1.8–3.6 mL/mmHg) versus 1.7 mL/mmHg (1.1–3.9 mL/mmHg) in PAH and PVH, respectively, which was not statistically significant, even with delineating differences in PAWP. There were no significant differences in Ees, Ea, CI, or Beta (β) as noted in Table 4.

**Table 4:** Echocardiographic and hemodynamic characteristics of PLWH with pulmonary arterial hypertension versus pulmonary venous hypertension.

	Pulmonary Arterial Hypertension	Pulmonary Venous Hypertension	P value
	N = 13	N = 14	
**Heart rate (bpm)**	74 (20)	74 (13)	0.99
**ePASP (mmHg)**	53 (33)	40 (23)	0.31
**EF (%)**	55 (50–60)	55 (50–60)	0.69
**RVOT VTI (m)**	0.16 (0.05)	0.15 (0.05)	0.85
**mPAAT (cm)**	111 (89–208)	128 (106–131)	1
**TAPSE (cm)**	1.8 (0.69)	2.0 (0.64)	0.6
**FAC (%)**	31 (13)	43 (15)	0.1
**MPAP (mmHg)**	32(28–49)	36(29–55)	0.65
**PAWP (mmHg)**	11 (9–13)	22 (18–33)	<0.01
**CO (L/min/m**^**2**^**)**	5.12 (1.16)	4.95 (2.13)	0.8
**PAC (mL/mmHg)**	2.5 (1.8–3.6)	1.7 (1.1–3.9)	0.54
**PVR (Woods Units)**	3.42 (3.0–7.5)	3.9 (1.7–5.4)	0.32
Ees (mmHg/mL)*	0.62 (0.46–0.97)	0.96 (0.46–1.2)	0.53
Ea (mmHg/mL)*	0.78 (0.53–1.2)	0.81 (0.52–1.9)	0.61
Ees/Ea*	0.84 (0.6–1.1)	0.76 (0.59–1.4)	1
Diastolic Stiffness Beta*	0.03 (-0.10–0.16)	0.08 (0.01–0.31)	0.15

Results reported as number (%), mean (standard deviation) or median (interquartile range). CI indicates cardiac output; Ea, arterial elastance; Ees, end systolic elastance; EF, ejection fraction; ePASP, estimated pulmonary artery systolic pressure; FAC, fractional area change; mPAAT, mean pulmonary artery acceleration time; mPAP, mean pulmonary artery pressure; PAH, pulmonary arterial hypertension; PAWP, pulmonary artery wedge pressure; PAC, Pulmonary artery compliance; PVR, pulmonary vascular resistance; RVOT VTI, right ventricular outflow tract velocity time integral; TAPSE, tricuspid annular plane systolic excursion.

*Single beat analysis was able to be calculated for 13 patients in the Pulmonary Arterial Hypertension group and 11 patients in the Pulmonary Venous Hypertension group.

HIV-PAH patients treated with vasodilator therapy (n = 9), there were substantial, but no significant reductions in the mean systolic pulmonary artery pressure (sPAP, 84 mmHg [81–101 mmHg] to 63mmHg [50-91mmHg], P = 0.10), mPAP 57 mmHg [52–64 mmHg] to 44mmHg [34–59 mmHg], P = 0.13), and improvement in cardiac output via Fick (CO 3.96 ±0.9 L/min to 4.82 ±1.4 L/min, P = 0.10) based on RHC pre- and post-therapy. By echocardiography pre- and post-therapy, there were non-significant reductions in ePASP (91±34 mmHg to 75±37 mmHg, P = 0.22) and mean TRV (4.4 ±0.9 m/s to 4.1± 1.0 m/s, P = 0.31). Repeat echocardiograms on routine follow up did not show further reductions in ePASP on patients with stable dosing of vasodilators.

### Survival and mortality in pulmonary hypertension

**Fig 1:**
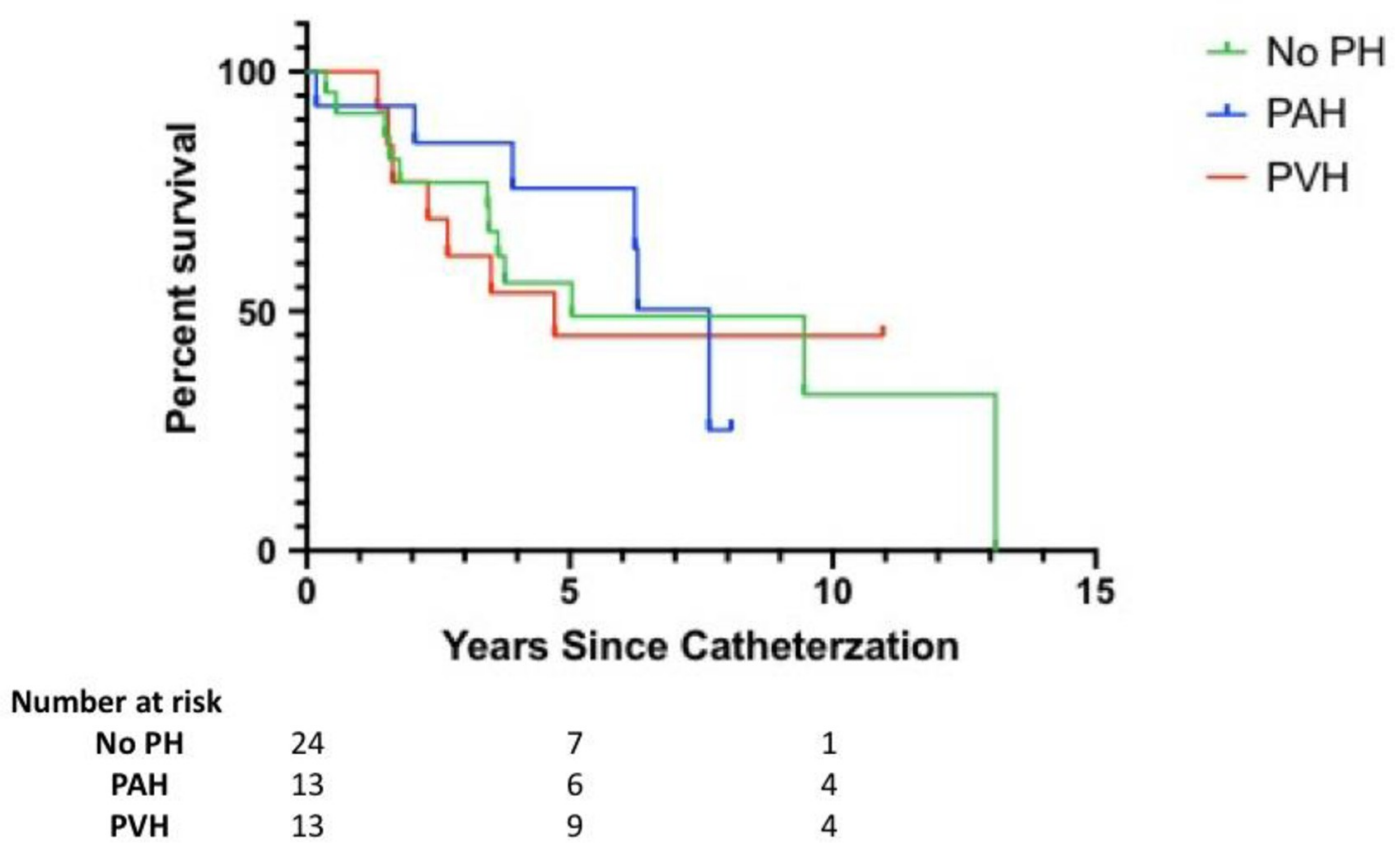
Kaplan Meir survival estimates. Survival between groups. PH indicates pulmonary hypertension; PAH, pulmonary arterial hypertension; PVH, pulmonary venous hypertension.

Follow-up data was available for 50 patients (24 No PH, 13 PAH, 13 PVH). The time from diagnosis of HIV to RHC to determine the presence or absence of PH on average was 11 years. Overall, the median survival in the entire cohort was 6 years. After diagnosis based on RHC, median survival was 8 years in PAH, 5 years in PVH, and 5 years in no PH (Fig 1), with no significant difference between groups (χ^2^ = 0.32, P = 0.85).

## Discussion

We evaluated echocardiographic and hemodynamic characteristics of PH within the setting of HIV. In our cohort, HIV-related PH appeared to be a mild-to-moderate with well-compensated RV function. Pertinent positive findings of PH included increased mPAP, PVR, Ees, Ea, and reduced RV FAC and RVOT VTI, while pertinent negative findings included no differences in ePASP, TAPSE, mPAAT, cardiac output, or Ees/Ea ratio. HIV patients developed both PAH and PVH with equal frequency. There were minimal echocardiographic or hemodynamic differences between PAH and PVH in this cohort. PH was associated with an elevated viral load and a lower CD4 count at the time of diagnosis, but no other demographic differences. We did not find significant differences in mortality in these patients with HIV based on PH status over a median follow-up of 6 years although numbers were small.

In this study, most patients were assumed as compliant with ART with generally well-preserved CD4 counts and well-controlled viral loads. However, we did find that HIV-PH was associated with CD4 counts <400 cells/mm^3^ at the time of diagnosis. The presence of a non-suppressed viral load did increase the odds of PH at the time of diagnosis, which may suggest the presence of viral activity being a trigger for the loss of proliferative control in the pulmonary vasculature. There has been at least one prior study of histopathology in HIV-PH which was unable to confirm an association between viral load and pulmonary vascular remodeling [20]. Our study suggests that in the current era, aggressive treatment of HIV may be associated with a lower incidence of PH which may not have been the case in the past [21, 22].

Interestingly, we found a similar prevalence of PAH and PVH in this cohort; however, we were unable to detect hemodynamic or echocardiographic differences (beyond the defining difference in PAWP). There is a paucity of evidence directly evaluating the presence of pulmonary venous hypertension in HIV. Early studies of HIV-related PH did not distinguish subtypes based on PAWP. The presence of diastolic dysfunction in HIV may be caused by an impaired diastolic reserve [6, 23], as well as the possible confounding effect of drug-induced metabolic syndrome due to chronic ART [24–26].

Determination of RV function is critical in PH as RV function is tightly linked to outcomes. HIV-related RV dysfunction may be independent of LV dysfunction [13] and viral activity may directly impair ventricular function [27, 28]. We evaluated RV function by echocardiography as well as by invasive hemodynamics with both standard measures available from clinical RHC and advanced measures of contractility (Ees), afterload (Ea), and ventriculo-arterial coupling (Ees/Ea). As noted above, RHC was able to differentiate subtypes of PH to greater accuracy based on mPAP, PAWP, and PVR despite an elevated ePASP in PH versus No PH groups on echocardiogram. Echocardiogram remains a crucial screening tool, with doppler derived measures gleaning insight to RV function that would be readily accessible, with the use of measures such as mPAAT, RVFAC, and RVOT VTI being able to differentiate the 2 groups. However, invasive hemodynamics are vital in PH, as PH can only be diagnosed by RHC to distinguish PAH from PVH and institute appropriate therapy.

The use of pressure-volume derived variables, Ea (RV afterload) and Ees (RV contractility), gives insight into the interaction between the RV and the pulmonary vasculature, evaluating its adaptation to load, i.e ventriculo-arterial coupling (Ees/Ea). In our cohort, there was a significant increase in Ees and Ea, with a nearly 2.5-fold increase in pressure generation compared to patients without PH. There was no change in the Ees/Ea ratio indicating compensated RV function (increased contractility to meet the increased load). This finding is not unusual given that Ees can increase up to 4 to 5-fold in response to an elevated PVR to maintain cardiac output [29]. The values of Ees/Ea were lower than the 1.5–2.0 ratio assumed to represent an optimal energy efficient state and may be due to the single beat method [14, 30, 31]. The increased RV afterload (Ea) seen in PH was in line with elevations in PVR and reductions in PAC, which reflect the steady state and pulsatile load, respectively, on the RV. Changes in pulmonary vascular stiffness have been associated with alterations in metabolism [32] and while no metabolomic data were available for the present study, the effect of HIV and PH on such markers would be an interesting future study.

Non-invasive evaluation of PH with echocardiography allows for screening by estimating probability of PH and allowing for further invasive testing. Screening is particularly important for at risk groups, such as those with HIV. We found that PLWH with PH had a moderate reduction in RV function, specifically demonstrated by FAC and RVOT VTI; however, TAPSE (a generalized measure of global RV function) was normal in the PH group. This discrepancy may indicate relatively preserved RV function, in keeping with the hemodynamic and survival data. We found that PLWH with PH had a moderate reduction in RV function, specifically demonstrated by the echocardiographic measures of FAC and RVOT VTI. Interestingly, TAPSE, a standard echocardiographic measure of RV function, was not different between groups. This may indicate that FAC and RVOT VTI are more sensitive to small changes in RV function than TAPSE. The RVOT VTI is a measure that takes into consideration outflow from the RV over the entire time course of systolic ejection [18, 33, 34]. The RVOT VTI findings suggest this measure may be useful as an early noninvasive marker of PH and may be used in conjunction with ePASP to non-invasively screen high-risk patients for PH.

Survival within HIV-related PH has been variable and difficult to discern given its relatively low prevalence. We found a median survival of 8 years in HIV-PAH which was not significantly different from the median survival of 5 years in HIV-PVH or from PLWH in this cohort without PH. This survival is better than that previously reported; however, it may be due to prior studies with sample populations that tend to have worse functional parameters (NYHA III-IV), study design heterogeneity, and PH not confirmed by RHC [3, 21, 22]. In our study, the longer survival time in PAH may be a consequence of lead time bias, given screening only occurs when symptoms arise, which may take an average of 2 years [4, 35] Additionally, in our cohort, PVR was less than 5 Woods units and RV function was preserved, suggestive of a significantly milder disease, which may be associated with better outcomes [36].

Antiretroviral and PH specific medications have may significant drug interactions, specifically non-nucleoside reverse transcriptase inhibitors (NNRTI) may result CYP-450 induced reduction of phosphodiesterase type 5 inhibitors (PDE5i) concentrations, with recommendations to monitor therapy based on clinical effect [37]. Whether or not these medication interactions may impact survival is unknown.

There are limitations to the present study. The retrospective nature of this study makes these findings hypothesis-generating and should be confirmed in larger prospective cohort given the relatively small sample size and limited power. We hypothesize that: 1) reductions in CD4 count or increases in viral load are associated with PH and worsening RV function; 2) echocardiographic measures of RV function such as RVOT VTI, S’, and mPAAT are sensitive early markers of RV dysfunction prior to generalized changes of global function such as TAPSE or estimated PASP. These measures may allow for early screening and diagnosis and do not require the presence of TR jet for evaluation. The single beat method for evaluating RV-PA coupling is an estimate that may not reflect measures derived from multiple simultaneous ventricular pressure and volume measurements [15]. Estimation of end systolic pressure for Ees and Ea using mPAP may lead to underestimation compared to PA conductance catheters [30]. CD4 counts and viral loads were only collected at the time of PH evaluation by RHC but would require interval evaluation to determine efficacy and compliance of HAART, as non-suppressed viral loads that persist after diagnosis of PH may impact RV function. CD4/viral counts require interval evaluation, as this may impact survival, and if mortality was related to PH or complications due to HIV (i.e. infection). Certain echocardiographic indices of RV and LV function such as S’, RV strain, and E’ were not obtained as these were not routinely measured clinically throughout the time period of the study.

## Conclusion

PLWH were diagnosed with both PAH and PVH which was associated with an elevated viral load and a lower CD4 count at the time of diagnosis. HIV-related PH was generally mild with well-compensated RV function with corresponding survival similar to those tested without PH. Further prospective studies in PLWH are warranted to determine the spectrum of PH in HIV and its associated RV adaptation and outcomes in the current era of highly effective ART.
